# Genetic variants underlying precancerous conditions of hepatocellular carcinoma

**DOI:** 10.1002/ijc.70092

**Published:** 2025-08-20

**Authors:** Jonathan Jaime G. Guerrero, Paolo C. Encarnacion, Mark Angelo S. del Rosario, Matthew Aldren S. Ora, Jiayan Zhou, Kin Israel Notarte, Wan‐Chun Li, Ching‐Wen Chang

**Affiliations:** ^1^ College of Medicine University of the Philippines Manila Philippines; ^2^ College of Public Health University of the Philippines Manila Philippines; ^3^ Graduate Institute of Metabolism and Obesity Sciences Taipei Medical University Taipei Taiwan; ^4^ Department of Industrial Engineering and Management Yuan Ze University Chung‐Li Taiwan; ^5^ Department of Biochemistry and Molecular Biology College of Medicine, UP Manila Manila Philippines; ^6^ Department of Medicine Stanford University School of Medicine Stanford California USA; ^7^ Department of Pathology Johns Hopkins University School of Medicine Baltimore Maryland USA; ^8^ Institute of Oral Biology, College of Dentistry National Yang Ming Chiao Tung University Taipei Taiwan; ^9^ Department of Dentistry, College of Dentistry National Yang Ming Chiao Tung University Taipei Taiwan; ^10^ Oral Medicine Innovation Center (OMIC) National Yang Ming Chiao Tung University Taipei Taiwan; ^11^ Department of Stomatology Taipei Veterans General Hospital Taipei Taiwan; ^12^ TMU Research Center for Digestive Medicine Taipei Medical University Taipei Taiwan; ^13^ Taipei Cancer Center Taipei Medical University Taipei Taiwan

**Keywords:** ALD, genetic variants, HCC, hepatitis, MASLD

## Abstract

Hepatocellular carcinoma (HCC) is the most common form of liver cancer, accounting for 80% of cases worldwide. While chronic hepatitis B and C infections remain primary risk factors, emerging evidence highlights the increasing contributions of metabolic dysfunction‐associated steatotic liver disease (MASLD) and alcohol‐associated liver disease (ALD) to HCC development. Genetic predispositions play a crucial role in modulating individual susceptibility to HCC, particularly through variants affecting viral persistence, lipid metabolism, and fibrogenesis. This review aims to summarize key genetic variants associated with precancerous conditions leading to HCC. The genetic risk factors, such as TP53 R249S mutant, TERT promoter mutations, and Wnt/B‐catenin pathway alterations, influence disease progression and treatment response in HCC subjects with chronic hepatitis B virus (HBV) and hepatitis C virus (HCV) infections. In MASLD‐related HCC, variants in PNPLA3, TM6SF2, and MBOAT7 modulate hepatic lipid metabolism and fibrosis. ALD‐associated HCC is influenced by polymorphisms in ADH1B, ADH1C, and ALDH2, which affect alcohol metabolism and oxidative stress. Additionally, inherited metabolic disorders, including Wilson's disease and hemochromatosis, further contribute to HCC susceptibility. Despite previous insights into HCC‐related genetic cues, challenges such as limited population‐specific data, lack of genetic screening programs, and ethical concerns regarding genetic tests hinder the translation of genetic discoveries into personalized HCC prevention strategies. Expanding population‐specific studies, improving genetic screening accessibility, and developing standardized risk prediction models will be crucial in shifting traditional medications toward a precision medicine setting for HCC management.

## Graphical abstract

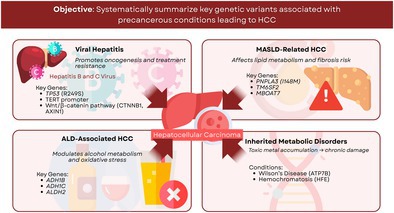

**Abbreviations**
AAT: alpha‐1 antitrypsin
ADH: alcohol dehydrogenase
AFP: alpha‐fetoprotein
AI: artificial intelligence
ALD: alcohol‐associated liver disease
ALDH: aldehyde dehydrogenase
CHB: chronic hepatitis B
CHC: chronic hepatitis C
DAA: direct‐acting antiviral
DCP: des‐gamma‐carboxy prothrombin
GSD I: glycogen storage disease type I
GSD Ia: glycogen‐6‐phosphatase deficiency
GSD Ib: glucose‐6‐phosphate translocase deficiency
G6Pase: glucose‐6‐phosphatase
HBeAg: hepatitis B envelope antigen
HBsAg: hepatitis B surface antigen
HBV: hepatitis B virus
HCC: hepatocellular carcinoma
HCV: hepatitis C virus
HFE: high Fe^2+^
HH: hemochromatosis
HLA: human leukocyte antigen
IFNL: interferon lambda
MASLD: metabolic dysfunction‐associated steatotic liver disease
PNPLA3: patatin‐like phospholipase domain‐containing protein 3
SNP: single nucleotide polymorphism
TERT: telomerase reverse transcriptase
TM6SF2: transmembrane 6 superfamily member 2
WD: Wilson's disease

## 1 INTRODUCTION

Liver cancer is the sixth most frequently occurring cancer and third leading cause of cancer‐related deaths worldwide.^1^ Age‐standardized prevalence rate of liver cancer shows a decrease globally, but with an observed region‐specific increase such as in America.^2^ In 2022, global incidence was estimated to be at 8.6 per 100,000 cases, with hepatocellular carcinoma (HCC) accounting for 75.5% of all cases.^3^

HCC is the dominant histological subgroup of liver cancer with higher incidence in East Asia and islands of the Asia‐Pacific.^4^ Asia accounts for more than 70% of HCC cases^5^ primarily due to the endemic presence of chronic hepatitis B and C infections, a strong risk factor for the development of the disease.^6^ East Asian countries of South Korea, Japan, and China, along with the Southeast Asian country of the Philippines, show a descending trend since 1978.^7^, ^8^ Gender‐wise, HCC is four‐fold greater in men than in women, especially in countries such as North and South Korea, Indonesia, and Vietnam.^9^

While hepatitis infections remain to be the main precancerous conditions of HCC, especially being endemic in East and Southeast Asia, the changing global epidemiology now highlights the significant contributions of other pathologic conditions such as metabolic dysfunction‐associated steatotic liver disease (MASLD), alcohol‐associated liver disease (ALD), and cirrhosis to the pathogenesis of HCC.^10^, ^11^, ^12^ The shift is particularly evident in regions with rapid urbanization, where lifestyle has also tipped the scale toward key drivers of MASLD, including obesity, type 2 diabetes, and hypertension.^10^, ^11^, ^12^ Furthermore, MASLD in the context of a high burden of hepatitis infection amplifies the disease progression, possibly creating a dual burden in many countries such as the Philippines and Vietnam, where hepatitis B virus (HBV) is endemic.

**FIGURE 1:**
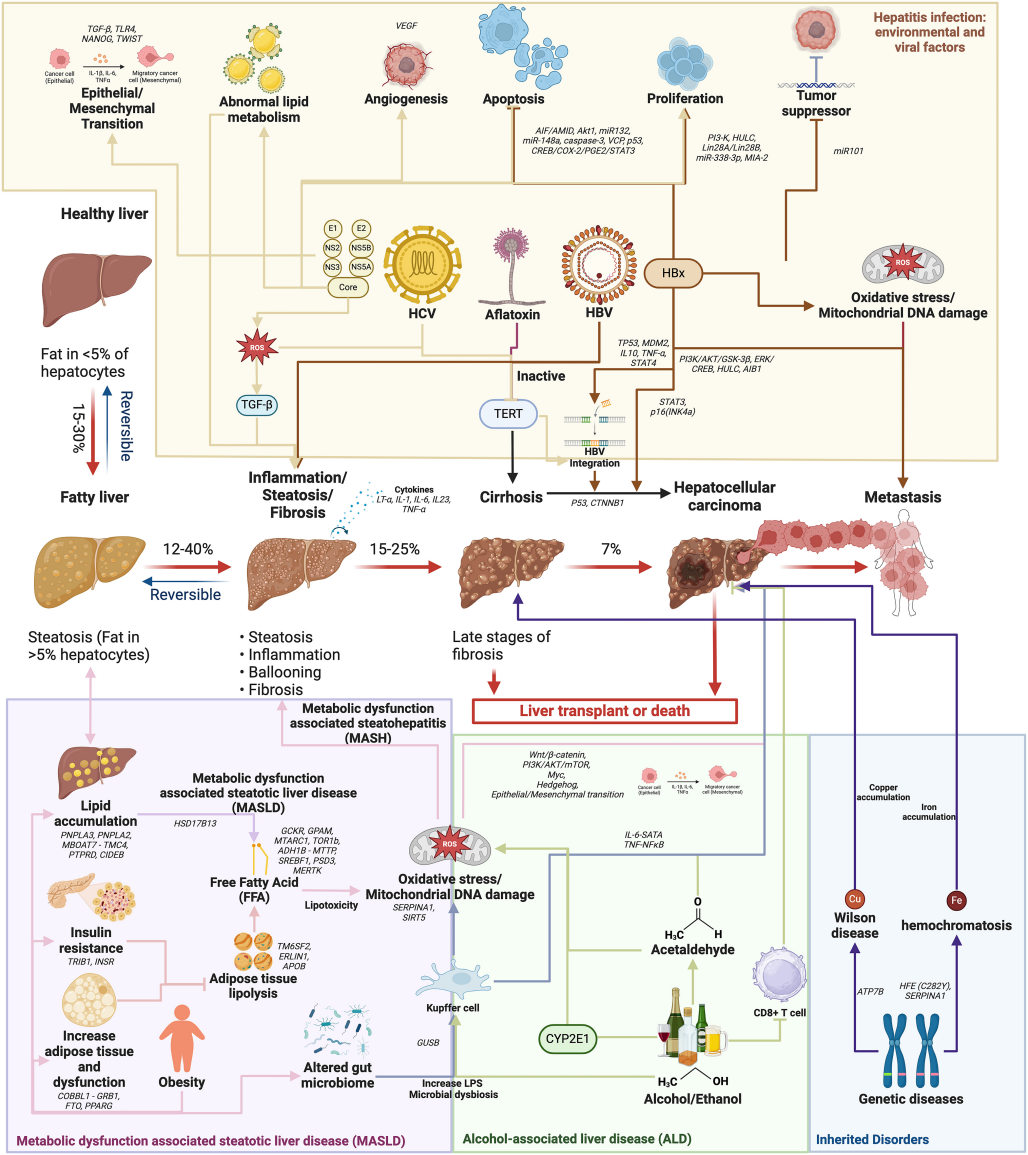
Mechanisms of hepatocellular carcinoma development from precancerous conditions. The progression of HCC is described using directional red arrows, which illustrate the various stages of the disease along with the corresponding percentage likelihood of progression to the next stage. Additional blue arrows indicate potential reversibility of certain conditions. Key mechanisms through which various precancerous conditions contribute to the progression of HCC include chronic hepatitis infection from environmental and viral factors (Yellow), metabolic dysfunction‐associated steatotic liver disease (MASLD) (Pink), alcohol‐associated liver disease (ALD) (Green), and inherited disorders (Blue). Each pathway highlights the roles of inflammation, oxidative stress, fibrosis, and genetic variants that predispose individuals to liver damage and neoplastic transformation. Potential genes were labeled in italics.

Of interest are the genetic variants which can significantly influence the pathogenesis of HCC (Figure 1). Variants in genes, such as Patatin‐like phospholipase domain‐containing protein 3 (PNPLA3) and Transmembrane 6 superfamily member 2 (TM6SF2), not only increase susceptibility to liver diseases but are also influenced by the rapid changes in the sociodemographic profile of countries worldwide. With the rising prevalence of MASLD, the mainstay risks of ALD, and the established risks of hepatitis infections, a better understanding of HCC associated genetic variants becomes critical to provide insights into disease prevention and progression as well as for a superior choice of therapeutic options. In this review, we seek to recap genetic variants of precancerous conditions of HCC—hepatitis infection, MASLD, ALD, and a few inherited disorders (Wilson's disease (WD), Hemochromatosis), and discuss the implications in both clinical and public health settings.

## 2 METHODOLOGY

To gather relevant literature for this review, a comprehensive search was performed using PubMed, Scopus, and Web of Science, covering publications from January 2000 to June 2024. The search focused on genetic variants associated with precancerous liver conditions leading to HCC. Keywords included combinations such as “hepatocellular carcinoma,” “HCC,” “genetic variants,” “polymorphisms,” “hepatitis B,” “von Gierke disease,” and “alpha‐1‐antitrypsin deficiency,” with Boolean operators used to refine the results.

We included peer‐reviewed articles, reviews, and meta‐analyses published in English that discussed the role of genetic polymorphisms in the development or progression for liver diseases known to precede HCC. Priority was given to studies involving human data or those with clear relevance to human disease. Articles were excluded if they were non‐English, inaccessible full texts, or did not contain substantive discussion of genetics in the context of HCC‐related precancerous conditions. Additional references were identified through manual screening of bibliographies of key studies.

## 3 GENETIC VARIANTS IN CHRONIC HEPATITIS B AND C INFECTIONS

Chronic hepatitis B (CHB) and hepatitis C (CHC) infections are significant global health challenges, affecting billions of individuals, leading to severe liver complications, including cirrhosis and HCC.^13^ The genetic variability of its vectors, HBV and hepatitis C virus (HCV),^14^, ^15^, ^16^ plays a crucial role in geographical distribution, transmission dynamics, pathogenesis, progression, and responsiveness to anti‐viral treatment. Both HBV and HCV exhibit significant genetic diversity in the genotype, subgenotype, and gene mutation levels, influencing disease outcomes, therapeutic strategies, and modes of transmission.

### 3.1 Hepatitis B

Based on antigenic determinants, HBV was initially categorized into nine serological subtypes. In 1988, the complete nucleotide sequences of 18 HBV strains revealed four distinct genotypes (A to D), defined by sequence differences of more than 8%.^17^, ^18^ Since then, more than 10 genotypes (A to J) have been identified globally, with a 4% sequence divergence threshold further classifying them into sub‐genotypes, including A1–A7, B1–B9, C1–C16, D1–D8, and F1–F4.^19^, ^20^ This genotypic diversity underpins variations in clinical presentation, progression, and treatment response.^21^, ^22^

HBV genotypes exhibit region‐specific prevalence. Genotype A predominates in North America, sub‐Saharan Africa, and parts of Europe.^23^, ^24^ Genotypes B and C are prevalent in East Asia and Southeast Asia, with genotype C particularly dominant in Korea and Japan.^25^, ^26^ Genotype D is widely distributed across the Mediterranean, Middle East, and South Asia.^27^, ^28^ Genotype F is primarily found in indigenous populations of South and Central America, while genotype H is localized to parts of Mexico.^29^, ^30^ The rare genotype G is commonly observed in co‐infection scenarios, often alongside genotype A, with cases sporadically reported in North America and Europe.^31^, ^32^

HBV genotypes influence the clinical course of infection and treatment outcomes. For example, genotype A is associated with higher rates of HBeAg (Hepatitis B envelope antigen) seroconversion and long‐term biochemical remission compared to genotype D.^20^, ^33^ HBsAg (Hepatitis B surface antigen) clearance is more evident in genotypes A and B than in genotypes C and D, with genotypes C and F linked to more severe liver disease and increased risk of HCC.^34^, ^35^ Patients with genotype C experience delayed HBeAg seroconversion and are more likely to be HBeAg positive at older ages than those with genotype B.^26^, ^36^ Moreover, genotype D poses a distinct risk for fulminant hepatitis.^25^, ^29^

Genotype‐specific mutations also affect disease progression and antiviral resistance. For instance, mutations in the precore and basal core promoter regions are prevalent in genotypes B and C, contributing to the development of HCC.^37^, ^38^ Similarly, genotype D is characterized by mutations that may exacerbate liver inflammation and fibrosis.^35^, ^39^ These findings underscore the need for genotype‐specific management strategies to optimize treatment efficacy and prevent complications.^19^, ^31^

### 3.2 Hepatitis C

HCV genome possesses extensive genetic variability, with different types of the virus showing up to a 33% difference across the entire viral genome.^40^ Genotype 1 is the most prevalent globally, as subtypes 1a and 1b are especially common in the United States, Europe, and Japan.^15^, ^41^, ^42^, ^43^ Subtype 1b is notably associated with severe liver conditions, including a higher incidence of cirrhosis and HCC.^15^, ^16^ It is also more frequently observed in patients with liver disease severe enough to require transplantation and those experiencing progressive graft injury. Another widespread genotype, Genotype 2, shows the most diversity in central and West Africa.^14^, ^41^ In North America, Europe, and Japan, subtypes 2a and 2b are prevalent,^42^, ^43^ while subtype 2c is commonly found in northern Italy.^15^

Genotype 3 ranks as the second most common genotype globally,^15^, ^16^ with a common presence in Northern Europe, South Asia, and Southeast Asia.^41^, ^43^, ^44^, ^45^ This genotype is associated with faster progression of liver disease, higher rates of steatosis, and an elevated risk of liver cancer.^40^ Additionally, genotype 3 presents certain pathological conditions, such as insulin resistance and complications in treatment with direct‐acting antivirals (DAAs).^16^, ^40^ Genotype 4 is primarily found in North Africa^14^ and the Middle East,^14^, ^46^, ^47^, ^48^, ^49^ whereas genotype 5 is mostly confined to South Africa, with occasional occurrences in diverse European regions.^41^ Genotype 6, along with its subtypes, is predominantly observed in Southeast Asia, including China, Hong Kong, and Vietnam.^41^, ^43^, ^50^

The genetic diversity and regional distribution of HCV genotypes carry significant implications for clinical practice.^15^, ^16^ The specific HCV genotype can influence disease progression, the likelihood of developing liver cancer, and the response to treatment. In particular, genotypes 1 and 3 are linked to a more rapid progression of liver disease and worse outcomes in HCV‐related HCC. Therefore, a deep understanding of HCV's genetic diversity is essential for guiding treatment decisions and managing the disease on a global scale.^15^, ^16^

### 3.3 Somatic mutations in hosts

The progression from viral infection to liver cancer is a complex process influenced by both viral factors and host genetic alterations. Among these, host somatic mutations are critical to determining HCC susceptibility. One of the most frequently observed somatic mutations in hepatitis virus‐related HCC involves the tumor suppressor gene TP53. This gene plays a pivotal role in maintaining genomic stability by regulating cell cycle arrest, apoptosis, and DNA repair.^51^ Mutations in TP53, such as the R249S variant, are particularly common in HBV‐endemic regions^52^, ^53^ and are often associated with exposure to a dietary carcinogen aflatoxin B1.^54^, ^55^ The coexistence of an HBV infection and TP53 mutation results in profound genomic instability, facilitating the accumulation of additional mutations that drive malignant transformation.^52^, ^53^ The loss of TP53 function is a significant step in the multistage process of carcinogenesis in many cancers,^51^ underscoring its role as a cornerstone in the pathophysiology of HCC. While TP53 mutations typically arise in fully developed hepatocellular carcinoma, rather than in precancerous lesions, they contribute substantially to late‐stage tumor progression.

Another critical pathway implicated in HCC is the Wnt/*β*‐catenin signaling cascade, which regulates cell proliferation and differentiation.^56^ Mutations in CTNNB1, the gene encoding *β*‐catenin, are commonly detected in HBV‐mediated HCC cases.^57^, ^58^ These mutations lead to the constitutive activation of the Wnt pathway, bypassing normal regulatory mechanisms and promoting tumorigenesis.^57^ Similarly, mutations in AXIN1, a negative regulator of the Wnt pathway, destabilize the *β*‐catenin destruction complex,^59^, ^60^ further amplifying oncogenic signaling. Similar to TP53 mutations, alterations in CTNNB1 and AXIN1 are generally found in established tumors and represent later events in hepatocarcinogenesis. The high prevalence of these mutations in HBV‐related HCC highlights the interplay between viral infections and host transcriptional changes in disrupting key cellular processes.

The reactivation of telomerase, a hallmark of cancer, is also a prominent feature of hepatitis virus‐related HCC.^61^, ^62^ Somatic mutations in the promoter region of the telomerase reverse transcriptase (TERT) gene, which encodes telomerase reverse transcriptase, are observed in a majority of HCC cases.^62^, ^63^ Unlike mutations in TP53 and CTNNB1, TERT promoter mutations are often detected in precancerous nodules and early‐stage lesions, suggesting their involvement in the initial steps of malignant transformation.^62^, ^63^ These mutations create novel transcription factor binding sites, leading to upregulated telomerase expression and enabling cells to bypass replicative senescence. The reactivation of telomerase is frequently driven by SP1 and ETS1 transcription factors, which bind to newly formed motifs at the mutated TERT promoter, enhancing its transcriptional activity.^62^, ^63^

In HBV‐related HCC, HBV DNA integration frequently targets the TERT promoter region. A genome‐wide analysis by Sung et al. identified TERT as the most common recurrent integration site, found in 23.7% of tumors, while Totoki et al. reported TERT integration in 22% of HBV‐positive HCC samples.^64^, ^65^ While TERT promoter mutations and HBV DNA integration are both mechanisms for telomerase activation, studies suggest that they may co‐occur within the same tumor; however, some evidence indicates mutual exclusivity in certain contexts.^62^, ^63^ In either case, the resulting overexpression of telomerase contributes significantly to the immortalization of hepatocytes and cancer development.

While many somatic mutations enhance susceptibility to HCC, some genetic alterations may reduce the likelihood of progression from hepatitis virus infection to cancer. For example, certain allelic variants of human leukocyte antigen (HLA) that play a critical role in modulating immune responses to viral infections^66^ are associated with enhanced antigen presentation and more effective clearance of hepatitis viruses, thereby reducing the risk of chronic infection and subsequent HCC.^67^ Similarly, polymorphisms in the interferon lambda genes, IFNL3 and IFNL4, influence the efficacy of antiviral immune responses. For instance, the rs12979860 CC genotype in IFNL3 is linked to improved clearance of HCV and a lower risk of HCC development.^68^, ^69^ These findings highlight the protective potential of specific genetic variants in mitigating the oncogenic effects of chronic hepatitis virus infections.

Collectively, the intricate interplay between somatic mutations and hepatitis virus infections underscores the multifaceted nature of HCC pathogenesis. Host genetic alterations, whether enhancing susceptibility or conferring protection, interact with viral factors to determine disease outcomes. For mutations such as those in TP53, CTNNB1, and TERT, the disruption of tumor suppressive mechanisms and activation of oncogenic pathways are key drivers of carcinogenesis. Importantly, the timing of these mutations, which are early for TERT promoter changes and late for TP53 and Wnt pathway mutations, shapes the sequence of molecular events leading to malignancy. On the other hand, protective genetic variants in genes like HLA and IFNL3 demonstrate the potential of the immune system to counteract viral‐induced oncogenesis in HCC subjects.

## 4 GENETIC VARIANTS IN METABOLIC DYSFUNCTION‐ASSOCIATED STEATOTIC LIVER DISEASE

A sedentary lifestyle increases the risk of a variety of medical conditions, including metabolic disorders. MASLD redefined the etiology and epidemiology of HCC globally. With the rapid growth of obesity, type 2 diabetes, and hypertension—key drivers of MASLD—it has been found that the rise of liver diseases acts as a significant precursor to HCC progression. While hepatitis infections remain a leading causative agent, MASLD is significantly playing a crucial role in promoting HCC progression, especially in countries where hepatitis infection is not endemic. Furthermore, MASLD also amplifies HCC risks in areas where hepatitis infection is widespread.

Key genetic polymorphisms have been implicated in the susceptibility, progression, and oncogenic transformation of MASLD. Most of these genetic variants influence pathways related to lipid metabolism, inflammation, and fibrogenesis, all of which are parallel to the developmental course of HCC,^70^, ^71^, ^72^, ^73^ as shown in Table 1.

**TABLE 1:** Genetic variants in MASLD which are associated with the development of HCC.

Gene	Variant	Mechanism in MASLD	Impact on development of HCC	References
PNPLA3	I148M	Reduces functionality of adiponutrin, a key enzyme in the export of fats from hepatocytes	Promote fibrosis directly through specific fibrogenesis pathways, and act indirectly through mediation of portal hypertension	[72, 75, 76, 77]
TM6SF2	E167K	Independently lead to liver fat accumulation, increased intracellular droplets, and total cholesterol, and reduced VLDL secretion	Promotes expression of inflammatory cytokines IL‐2 and IL‐6, which may accelerate the progression of HCC; as well as affect cell cycle by upregulating CyclinD1, P53, and Rb and downregulating P27	[78, 79, 80]
GCKR	Pro446Leu	Increased circulating triglycerides and fibroblast growth factor 2 increases hepatic lipogenesis	Facilitates the transition from steatosis to fibrosis, increasing carcinogenesis risk	[81]
	P446L	Loss of function of GCKR, thus increasing glucose phosphorylation and fatty acid synthesis in the liver		[82, 83]
	rsI260326	Accumulation of fat in the liver alongside large VLDL and triglycerides level	Predisposes to overt fibrosis	[84, 85]
SREBF2	rs133291	Increased lipogenesis and hepatic steatosis	Predisposes to liver fibrosis and malignancy and malignant transformation	[86]
LEPR	Q223R, Lys109Arg, Gln223Arg	Impaired leptin signaling promoting chronic inflammation and metabolic dysfunction	Amplifies fibrogenesis and chronic inflammation, fostering an environment of oncogenesis	[18, 87]
APOB	c.6718A>T, K2240X	The nonsense mutation produces a truncated form of APOB which causes impaired secretion of VLDL in the liver, ultimately leading to accumulation of lipids in the liver	Predisposes to chronic inflammation, liver fibrosis, and malignancy	[88]
TERT	c.2062 C>G (Glu668Asp)	Hepatocellular accumulation of lipids and iron due to hepatocellular senescence	Predisposes to steatosis, fibrosis, and hepatocellular carcinogenesis	[89]
MBOAT7	rs641738 C >T	Impaired lipid metabolism—shift in phosphatidyl‐inositol composition—in liver cells which promotes hepatocellular accumulation of lipids and inflammation of the liver	Predisposes to fibrosis, chronic inflammation, and hepatocellular carcinogenesis	[90]
HSD17B13	rs72613567 T/TA (major allele; in contrast to minor allele which has been associated with reduced risk for MASLD)	Impaired metabolism of steroid hormones and fatty acids resulting to accumulation of lipids in hepatocytes	Predisposes to fibrosis, chronic inflammation, and hepatocellular carcinogenesis	[91]
CELSR2‐PSRC1‐SORT1	rs599839 A >G	No significant effect on hepatic fat accumulation or inflammation but has been found to increase risk of developing HCC in patients with MASLD	Predisposes to uncontrolled proliferation and survival of hepatocytes due to overexpression of PSRC1	[92]
TM6SF2	E167K	Impaired VLDL secretion causing increased hepatic accumulation of triglycerides	Predisposed to hepatic fibrosis and cirrhosis	[93]

Other than the disease‐associated promoting cues, some gene variants were reported serving as protectors on MASLD or MASLD‐associated HCC. The missense variant of the Mitochondrial Amidoxime Reducing Component 1 (MTARC1 p.A165T) has been shown to be protective against hepatic fibrosis in obese individuals and buffers the effects of the PNPLA3 p.I148M allele.^74^ Carriers of rs7261357:TA, a gene variant of Hydroxysteroid 17‐Beta Dehydrogenase 13 (HSD17B13), have been shown to correlate with decreased hepatic fibrosis and downregulation of the expression of inflammatory gene expression.^75^ It is noteworthy that, to date, the role of the genetic variant of HSD17B13 is still under debate for its association with reduced risk of MASLD and steatohepatitis.^76^

## 5 GENETIC VARIANTS IN ALCOHOLIC LIVER DISEASE AND INHERITED METABOLIC DISORDERS

### 5.1 *Adh* and *Aldh* genes

Alcohol abuse accounts for a major global issue, currently ranked as the eighth leading risk factor for premature mortality and disability.^94^ Epidemiological statistics indicate a global rise in liver disorders strongly linked to alcohol abuse, wherein over 90% of individuals who engage in chronic alcohol use develop steatosis, or fatty liver^95^—the initial stage of liver pathological transformation toward HCC. This condition can progress to more severe stages, including inflammation (i.e., steatohepatitis), fibrosis, cirrhosis, and ultimately, liver cancer.^96^ Interestingly, some individuals seem more at risk for the progression of these diseases. Indeed, as shown in Table 2, numbers of genetic variants contribute to susceptibility to ALD‐mediated carcinogenesis.

**TABLE 2:** Genetic variants in alcoholic liver disease and inherited metabolic disorders.

Gene	Variant	Mechanism in liver disease	Impact on Development of HCC	References
ADH1B	ADH1B2 (rs1229984, p.Arg48His)	Higher alcohol metabolizing activity compared to wild‐type allele	Association with HCC remains contentious; polymorphisms in the human adh gene are not considered relevant in HCC development	[97, 98, 104, 105, 106, 107, 108, 109]
ADH1C	ADH1C2 (rs698, p.Ile350Val)	Reduced activity (~1.5–2 fold) compared to ADH1C1	Combined with other variants may increase risk of alcoholic cirrhosis; HCC impact unclear	[98]
ALDH2	ALDH22 (rs671, p.Glu487Lys)	Increased circulating triglycerides and fibroblast growth factor 2 increases hepatic lipogenesis	Conflicting evidence: some studies report minimal association with HCC; others report increased risk especially in heterozygotes; acetaldehyde accumulation promotes hepatocarcinogenesis	[104, 105, 106, 107, 110, 111, 112, 113, 114, 115, 116]
ATP7B	p.His1069Gln (Ashkenazi Jewish, European); p.Thr935Met; p.Arg778Leu (East Asian)	Disrupted copper excretion causing hepatic copper accumulation; oxidative stress from copper and iron promotes hepatocyte damage and cirrhosis	HCC is rare despite cirrhosis; metal‐induced oxidative stress may promote carcinogenesis	[118, 119, 120, 121, 122, 123, 124, 125, 126]
HFE	C282Y	Abnormal iron absorption leads to iron overload in the liver, triggering inflammatory response and mutagenic hydroxyl radicals	20%–69% progress to cirrhosis; ≥20‐fold increased risk, even without cirrhosis	[127, 129, 130, 131, 132]
SERPINA1	Z (Glu342Lys); Siiyama (Ser53Phe); Mmalton (Phe52del)	Impaired AAT production leads to accumulation of misfolded proteins in the liver, inducing chronic stress and cellular injury	Increased HCC risk, including in non‐cirrhotic cases; cell cycle disruption through cyclin D1 and MCAM	[133, 134, 135, 136, 137, 138]
G6PC	G727T (Japanese); G327A (Chinese)	Deficient glucose‐6‐phosphatase causes metabolic disturbances which subsequently cause hepatic adenoma formation	Hepatocellular adenomas may undergo malignant transformation into HCC	[139, 140, 141, 142, 143, 144, 145]
CTNNB1	Del7L–131L; del21G‐98M + ins21CC	No noted direct effect on development of von Gierke disease	Adenomas may undergo malignant transformation	[144]

Genetic factors govern alcohol metabolism and significantly influence the etiology of ALD and liver cancer progression. Host genetic factors, such as variations in the activity and functionality of alcohol‐metabolizing enzymes, and environmental factors, such as alcohol consumption levels and overall nutrition, contribute considerably to this regard.^97^ In humans, the xenobiotic machinery that regulates alcohol metabolism is mediated mainly by alcohol dehydrogenases (ADHs) and aldehyde dehydrogenases (ALDHs). From this superfamily of enzymes, three single nucleotide polymorphisms (SNPs) emerge as the most prevalent and well‐studied variants among the Asian population (mainly East Asians) that are linked to ALD and liver cancer risk: (1) the ADH1B*2 variant (rs1229984: c.254G>A, p.Arg48His); (2) ADH1C*2 variant (rs698: c.1418A>G, p.Ile350Val); and (3) the ALDH2*2 variants (rs671: c.1606G>A, p.Glu487Lys). The ADH1B*2 allele is associated with higher alcohol metabolizing activity relative to the wild‐type ADH1B*1 allele. Conversely, the ADH1C*2 allele exhibits approximately 1.5‐ to 2‐fold reduced activity compared to the ancestral ADH1C*1 phenotype. Additionally, the presence of one or both ALDH2*2 alleles can severely decrease enzymatic activity to 5%–20% compared to those with the wild‐type allele.^98^

To date, the existing data on ADH and ALDH gene mutations and their association with ALD and HCC risk remain contentious. While some studies suggested that ADH polymorphisms are not associated with ALD susceptibility,^97^, ^98^, ^99^ others dispute these findings.^98^, ^100^, ^101^, ^102^, ^103^ In contrast, there is a consensus that polymorphisms in the human *adh* gene are not relevant in the development of HCC.^104^, ^105^, ^106^, ^107^, ^108^, ^109^ Meanwhile, available data surrounding *ALDH* gene mutations and its association with ALD and HCC risk, so far, remain a subject of ongoing debate. The majority of the East Asian studies argue that ALDH2 mutants had limited risk for HCC onset.^104^, ^105^, ^106^, ^107^, ^110^, ^111^, ^112^ In fact, based on a study involving 102 patients with HCC and 125 healthy controls, Takeshita et al.^104^ identified chronic heavy drinking as a major environmental risk factor for HCC. However, the ALDH2*1/*2 and ADH2 genotypes showed minimal association with HCC development. Several years thereafter, many researchers challenged Takeshita's perspective. Munaka et al.^113^ found an increased risk among individuals with at least 1 copy of the ALDH2*2 allele, while Tomoda et al.^114^ found an increased risk for heterozygous individuals only. Meanwhile, Yu's^115^ study demonstrates that the metabolic capacity of acetaldehyde in ALDH2*1/*2 gene drinkers is so compromised that it results in a rapid accumulation of acetaldehyde. This accumulation leads to increased levels of ROS and DNA damage, ultimately promoting hepatocarcinogenesis. Similarly, Abe et al.^108^ argued that ALDH2 polymorphism is closely related to ethanol consumption patterns, and both ALDH2 polymorphism and duration of alcohol use influence the development of HCC in patients with alcoholic liver cirrhosis, and Ye et al.^116^ reported that ALDH2 polymorphism is linked to significant variations in HCC susceptibility. Furthermore, it was also recently reported that the combined genotypes of ADH1B rs1229984, ADH1C rs698, and ALDH2 rs671 that do not lead to acetaldehyde accumulation are associated with an increased risk of alcoholic cirrhosis.^98^ Lastly, a meta‐analysis of He et al.^117^ seems to contradict that ALDH2 polymorphism increases the risk of developing alcoholic cirrhosis. While the ALDH2 rs671 genotype that leads to acetaldehyde accumulation was previously thought to predispose individuals to reactive aldehyde and, thus, increase the risk for alcoholic cirrhosis, the accumulation of acetaldehyde and its associated unpleasant symptoms actually discourage alcohol consumption; thus, the genotype is, in fact, a protective factor rather than a risk.

### 5.2 Wilson's disease

WD, also known as hepatolenticular degeneration, is an autosomal recessive disorder of copper metabolism causing accumulation of copper in the liver, brain, and other organs. This disorder has diverse clinical manifestations, with hepatic involvement being a key feature. It often presents with hepatic dysfunction and/or hepatocellular damage, which can lead to liver cirrhosis.^118^ The disorder in copper metabolism is explained by pathogenic variants in the ATP7B gene, which encodes a copper‐transporting ATPase involved in the excretion of excess copper into bile, thereby leading to copper accumulation.^119^ Furthermore, the diversity in clinical manifestations of WD can be accounted for by the variety of mutations, with geographic and ethnic predilection, of the abovementioned gene, including missense mutations which disrupt protein folding and nonsense mutations which render protein function completely lost.^120^ In a genetic epidemiological study by Wallace and Dooley,^121^ 732 variants of ATP7B have been implicated in WD; 400 of those have been identified as missense variants while 279 as complete or near‐complete loss of function. Moreover, p.His1069Gln has been identified as the most prevalent pathogenic variant of ATP7B in the Ashkenazi Jewish population and also in the European population, while p.Thr935Met and p.Arg778Leu are the most prevalent in East Asian populations.^121^

HCC is a well‐known complication of cirrhosis; however, despite WD's propensity to lead to liver cirrhosis, it is considered only a rare complication in this context.^122^, ^123^ The low incidence of HCC in patients with WD may be attributed to the shortened life expectancy in untreated patients, which limits the time for carcinoma to develop.^124^ The exact mechanism of how HCC develops in patients with WD has not been completely elaborated yet. Kato et al.,^125^ however, suggest that the hepatic fibrosis and carcinogenic predisposition in WD are related to metal‐induced oxidative stress, that is, the hepatic accumulation of iron and copper promotes the formation of reactive radicals, which drive lipid peroxidation and incur cellular and DNA damage. This is consistent with a case report by Reyes^126^ of a 59‐year‐old white man with WD diagnosed with HCC, where accumulation of excess copper in his liver, along with the presence of concurrent cirrhosis, has been suggested as the most probable cause of HCC formation.

### 5.3 Hemochromatosis

Hemochromatosis (HH) is a collection of genetic disorders characterized by abnormal iron absorption, resulting in the gradual accumulation of excess iron, especially in the liver. The majority of these disorders (Hemochromatosis Type 1) are caused by mutations in high iron (HFE) genes. Common genotypes include C282Y homozygosity, C282Y/H63D compound heterozygosity, and other HFE‐related variants such as S65C.^127^ Rarer genotypes involve mutations in non‐HFE genes, such as HJV (c.‐6C>G), HAMP (‐72C>T, R59G, G71D), TFR2 (no specific mutations identified), SLC40A1 (Q248H), and FTL (L55L).^128^

Cirrhosis has been found to develop in 20%–69% of individuals with homozygous HFE C282Y mutation.^130^ Furthermore, hemochromatosis significantly increases the risk of HCC, with studies reporting at least a 20‐fold elevated risk, which may be explained by the hepatic iron overload that often triggers an inflammatory response and capacitates the formation of mutagenic hydroxyl radicals. Additionally, these highly reactive oxygen species promote lipid peroxidation, which may damage cellular components including DNA.^130^ The hepatic inflammation closely linked to the development of cirrhosis, together with the aforementioned DNA damage, predisposes individuals with hemochromatosis to carcinogenesis, a risk particularly pronounced in genotypes with HFE gene mutation, specifically the C282Y mutation.^129^, ^130^, ^131^ Moreover, even in the absence of cirrhosis, patients with HFE‐associated HH have an increased risk of HCC, further suggesting a carcinogenic role for hepatic iron deposition, primarily mediated by DNA damage and lipid peroxidation induced by iron‐driven oxidative stress.^132^

### 5.4 Alpha‐1 antitrypsin deficiency

Alpha‐1 antitrypsin (AAT) deficiency is a genetic disorder characterized by impaired production of the AAT, which is explained by mutations in the SERPINA1 gene, which codes for AAT. Over 90 genetic variants of AAT have been identified.^133^ Among these, Z (Glu342Lys), Siiyama (Ser53Phe), and Mmalton (Phe52del) are most strongly associated with increased HCC risk.^134^, ^135^

The increased risk of developing HCC in AAT deficiency arises from the underlying mechanism of the condition—improper synthesis of AAT, which leads to misfolded AAT proteins unable to exit the liver, causing their accumulation. The accumulation of such misfolded proteins induces chronic cellular stress that activates mitochondrial autophagy; however, in severe cases of AAT deficiency, this response becomes overwhelmed, resulting in mitochondrial injury contributing to hepatocellular damage.^136^ This hepatic accumulation also causes hepatic injury by virtue of a gain‐of‐toxic function mechanism. The damages incurred then initiate a hepatic inflammatory response that induces apoptosis, leading to chronic hepatocellular death and regeneration, which progressively results in fibrosis, cirrhosis, and ultimately HCC.^136^, ^137^ Additionally, the accumulation of misfolded AAT proteins triggers the expression of cyclin D1 and melanoma cell adhesion molecule (MCAM) gene, both of which are involved in regulating cell growth and promoting tumor development. This contributes to the formation of HCC. Furthermore, this mechanism may explain why individuals with AAT deficiency still have an increased risk for HCC even in the absence of cirrhosis.^139^

The overall risk of HCC in individuals with AAT deficiency, however, remains unclear due to limited studies. The prevalence of cirrhosis among affected individuals varies widely from 2% to 43%, with age being a major contributing factor. Lastly, despite evidence of an increased risk for HCC, its prevalence in association with AAT deficiency is still unclear.^137^

### 5.5 Von Gierke disease

von Gierke disease, also known as glycogen storage disease type I (GSD I), is an autosomal recessive disorder of carbohydrate metabolism that leads to glycogen accumulation.^139^ It is caused by glycogen‐6‐phosphatase (G6Pase) deficiency (GSD Ia) and deficiency of glucose‐6‐phosphate translocase (GSD Ib), both of which lead to hypoglycemia and lactic acidosis.^140^ The former is caused by mutations in the G6PC gene, which codes for glucose‐6‐phosphatase, while the latter is caused by mutations in the SLC37A4 gene, which codes for the glucose‐6‐phosphate translocase.^141^ As of writing, the Human Gene Mutation Database has identified 170 mutations in the G6PC gene, including 50 pathogenic missense mutations, 2 nonsense mutations, and 2 codon deletions.^142^

The abnormal glycogen metabolism in von Gierke disease triggers a variety of metabolic disturbances, conferring harm to the liver, which may cause hepatomegaly.^140^ Hepatic involvement in von Gierke disease often results in hepatocellular adenomas, some of which may undergo malignant transformation into HCC.^143^ Nakamura et al.^144^ reported the first documented case of HCC in GSD Ia associated with the G6PC variant G727T, which is common among Japanese individuals and is believed to cause splicing defects in G6Pase. Although the association between this mutation and HCC has not yet been fully established, both G727T and G327A (which is common among Chinese individuals) variants of G6PC have been subsequently detected in von Gierke disease patients with HCC.^145^ Mutations in the CTNNB1 gene, which encodes β‐catenin, have also been implicated in HCC development in von Gierke disease. For instance, Cassiman et al.^146^ identified two CTNNB1 mutations, Del7L‐131L and del21G‐98M + ins21CC, in a case of GSD I with malignant transformation of hepatocellular adenomas.

## 6 DISCUSSION

Despite significant advancements in understanding the genetic underpinnings of HCC, several gaps and challenges remain in integrating genetic insights into clinical and public health practice. One of the major gaps is the limited availability of large‐scale, population‐specific genetic studies, particularly in low‐resource regions with high HCC prevalence, such as southeast Asia. Most genetic studies on HCC risk have been conducted in Western or East Asian populations, leaving a knowledge gap regarding genetic variations that may be unique to populations in the Philippines and other parts of Southeast Asia.

Predictive models have become essential for identifying individuals at high risk or for assessing disease prognosis. These models leverage clinical, genetic, and molecular data to provide individualized risk assessments and guide therapeutic strategies. Early models primarily relied on clinical parameters such as age, sex, liver function tests, and the presence of chronic HBV or HCV infection.^147^ For HBV‐related HCC, viral load and specific genotypes have been pivotal in stratifying risk, while liver fibrosis staging, determined through liver biopsy or non‐invasive techniques like transient elastography, has proven to be a strong predictor in HCV‐associated cases.

Advancements in genomics and biomarker research have enhanced the accuracy of predictive models by incorporating molecular data. For instance, the integration of serum biomarkers like alpha‐fetoprotein (AFP) and des‐gamma‐carboxy prothrombin (DCP) has improved early detection and risk prediction. Furthermore, host genetic variations, including mutations in TP53 and TERT promoter regions, are now recognized as significant contributors to HCC susceptibility and are being incorporated into models. In parallel, polymorphisms in immune response genes, such as IFNL3 and HLA, have been associated with differential outcomes in hepatitis virus infections, further refining risk assessments.

Machine learning and artificial intelligence (AI) approaches are driving the development of next‐generation predictive models. By analyzing large datasets, these tools can identify complex patterns and interactions among clinical, genetic, and environmental factors that traditional methods might overlook. For example, AI‐based algorithms have been employed to analyze imaging data, such as CT and MRI scans, alongside clinical records to predict HCC development or recurrence more accurately. Moreover, multi‐omics approaches that integrate transcriptomic, proteomic, and metabolomic data are emerging as powerful tools for identifying novel biomarkers and improving model precision.

Despite these advancements, challenges remain in the application of predictive models for hepatitis virus‐related HCC. Variability in data quality, population heterogeneity, and limited access to advanced diagnostic tools in resource‐constrained settings can impact model reliability and generalizability. Furthermore, ethical considerations surrounding the use of genetic data and AI‐driven predictions need to be addressed to ensure equitable and responsible implementation. Nonetheless, the ongoing refinement of predictive models, coupled with technological innovations, holds great promise for improving the early detection and management of hepatitis virus‐related HCC, ultimately reducing its global burden.

Globally, approximately 80% of HCC cases are attributable to chronic HBV or HCV infections, with HBV alone accounting for around 360,000 HCC cases annually, representing 55% of the total.^147^ Although HCV is less common, it poses a greater risk, increasing the likelihood of HCC by 15–20 times compared to HBV. For individuals with HCV‐related cirrhosis, the annual incidence of HCC ranges from 2% to 4%.

These epidemiological findings represent a significant gap in the representation of non‐viral HCC etiologies, particularly MASLD and ALD, in genetic research. Historically, much of the focus on HCC has centered on HBV and HCV infections, given their well‐established roles in liver cancer. However, with the global decline in viral hepatitis due to vaccination and antiviral therapies, MASLD and ALD are emerging dominant risk factors, particularly in urbanized populations with rising obesity and metabolic syndrome rates. Despite this shift, genetic research on MASLD‐ and ALD‐related HCC remains relatively limited, and most predictive models still prioritize viral hepatitis over metabolic risk factors. This gap highlights the need for further studies exploring how genetic variants interact with obesity, insulin resistance, and lipid metabolism in driving liver cancer progression.

Furthermore, the integration of genetic biomarkers into routine clinical practice also presents challenges. While genetic testing for some conditions, such as HFE mutations in hemochromatosis, is available, widespread implementation of comprehensive genetic screening for HCC remains impractical in many healthcare systems, especially in low‐ and middle‐income countries. The high cost of genetic testing, limited access to advanced sequencing technologies, and lack of trained genetic counselors can be prohibitive and exacerbate the scenario.

### 6.1 Future directions

To effectively reduce the global burden of HCC, it is imperative to bridge the gap between genetic research, clinical application, and public health strategies. While significant progress has been made in identifying genetic variants that influence HCC risk, critical challenges remain in integrating these insights well into accessible healthcare, particularly in low‐resource settings. The underrepresentation of non‐viral etiologies, the complexity of gene–environment interactions, and the lack of standardized genetic screening guidelines hinder the development of precise, population‐specific risk models. Furthermore, ethical concerns regarding genetic data privacy and disparities in health care access must be urgently addressed to ensure that advancements in genetic research benefit all individuals equitably.

Moving forward, a multidisciplinary, collaborative approach—encompassing genomics, clinical medicine, AI, and public health—will be essential in transforming genetic discoveries into actionable interventions. Expanding population‐specific studies, integrating genetic data into predictive models, and improving accessibility to genetic screening are critical steps. By leveraging genetic insights, we can then shift to a proactive, precision‐based strategy for HCC prevention, early detection, and treatment.

## AUTHOR CONTRIBUTIONS

**Jonathan Jaime G. Guerrero:** Conceptualization; investigation; validation; writing – original draft; writing – review and editing; data curation. **Paolo C. Encarnacion:** Investigation; writing – original draft; writing – review and editing; data curation. **Mark Angelo S. del Rosario:** Writing – original draft; investigation; data curation. **Matthew Aldren S. Ora:** Investigation; writing – original draft; data curation. **Jiayan Zhou:** Investigation; visualization; software; data curation. **Kin Israel Notarte:** Investigation; visualization; software; data curation. **Wan‐Chun Li:** Conceptualization; validation; funding acquisition; supervision; writing – review and editing. **Ching‐Wen Chang:** Conceptualization; validation; supervision; writing – review and editing; funding acquisition.

## FUNDING INFORMATION

This study was supported by the National Science and Technology Council of Taiwan (NSTC113‐2311‐B‐038‐001 to CWC and NSTC113‐2314‐B‐A49‐017 to WCL), Taipei Medical University‐National Taiwan University Hospital Joint Research Program (113‐TMU092, 114‐TMU172), Taipei Medical University (TMU111‐AE1‐B44), and Taipei Medical University–Shuang Ho Hospital, Ministry of Health and Welfare (112TMU‐SHH‐22) to CWC.

## CONFLICT OF INTEREST STATEMENT

Authors declare no competing interest in the conduct and publication of this research.
